# Assessment of kinesiophobia in ulcerative colitis: a case-control study highlighting functional and psychological implications

**DOI:** 10.1590/1806-9282.20252276

**Published:** 2026-07-17

**Authors:** Óscar Fernando Sarmiento-Rodríguez, Federico Argüelles-Arias, Ricardo Becerro-de-Bengoa-Vallejo, Marta Elena Losa-Iglesias, Juan Gómez-Salgado, Natalia Tovaruela-Carrión, Miguel Ángel Saavedra-García, Daniel López-López

**Affiliations:** 1 Universidade da Coruña, Industrial Campus of Ferrol, Faculty of Nursing and Podiatry, Department of Health Sciences, Research, Health and Podiatry Group – Ferrol, Spain.; 2 Universidad de Sevilla, Hospital Universitario Virgen Macarena de Sevilla, Faculty of Medicine, Digestive Service – Sevilla, Spain.; 3 Complutense University of Madrid, Faculty of Nursing, Physiotherapy and Podiatry – Madrid, Spain.; 4 Universidad Rey Juan Carlos, Faculty of Health Sciences – Madrid, Spain.; 5 Universidad de Huelva, Department of Sociology, Social Work and Public Health – Huelva, Spain.; 6 Universidad Espíritu Santo, Safety and Health Postgraduate Program – Guayaquil, Ecuador.; 7 University of Seville, Faculty of Nursing, Physiotherapy and Podiatry – Seville, Spain.; 8 Universidade da Coruña, Department of Physical Education and Sports, Grupo INCIDE – Oleiros, Spain.

**Keywords:** Kinesiophobia, Ulcerative colitis, Fear of movement, Quality of life, Physical activity

## Abstract

**OBJECTIVE:**

Idiopathic colitis is a chronic disorder, and the pain, fatigue, and anxiety associated with this condition may promote the evolution of kinesiophobia, a fear of movement that limits physical activity and worsens quality of life. However, this relationship has been understudied. Therefore, the aim of this research was to compare kinesiophobia scores in ulcerative colitis subjects to those in healthy controls.

**METHODS:**

In this case-control study, 106 subjects participated; 53 healthy participants were selected from private podiatry clinics; and 53 patients with ulcerative colitis were selected from the Virgen Macarena University Hospital and the Association of Crohn's and Ulcerative Colitis Patients of Seville (ACCU) Seville association. Each participant received the abbreviated Spanish version of the Fear of Movement Scale, known as Tampa Scale for Kinesiophobia.

**RESULTS:**

Subjects with ulcerative colitis had higher total Tampa Scale for Kinesiophobia scores compared to healthy subjects (24.98 ± 8.01 vs. 21.94 ± 6.18; p=0.016). Moderate to severe kinesiophobia was present in 49.1% of the ulcerative colitis patients, compared with 30.2% in the control group. The ulcerative colitis group had a significantly higher percentage of participants with severe kinesiophobia (11.3 vs. 1.9%).

**CONCLUSION:**

These findings highlight the importance of early assessment of fear of movement to support interventions aimed at improving physical activity participation, functional capacity, and overall quality of life in this population.

## INTRODUCTION

Kinesiophobia is a disproportionate, irrational, and disabling fear of a certain physical movement, originating from a perception of fragility associated with that activity or from the fear of suffering a new painful injury^1^. It also includes fear of the symptoms of tiredness or exhaustion and of the physical or even mental discomfort that may appear with performing that movement, such that psychological defense mechanisms appear that cause the repression, inhibition, or denial of the need to perform said movement^2^.

Ulcerative colitis (UC) is a chronic intestinal disease, which affects the innermost layer of the mucosa of the large intestine. It has a relapsing-remitting course and presents with abdominal pain, rectal bleeding, tenesmus, diarrhea, and debilitating asthenia. In addition to intestinal manifestations, UC is frequently associated with extraintestinal symptoms, including musculoskeletal pain and joint involvement, which may contribute to physical limitations and reduced mobility^3-5^.

Physical activity can help reduce the physical and psychosocial burden of the disease, as it helps decrease stress and depression, regulates systemic inflammation, and promotes social relationships and quality of life, which could have a positive effect on the course of UC^6,7^. However, many patients reduce or avoid physical activity due to symptoms such as pain, fatigue, fear of symptom exacerbation, or fear and embarrassment of potential involuntary defecation. Therefore, although physical activity is beneficial for these patients, there are significant limitations and barriers to its implementation due to the recurrent nature of UC^8,9^.

Although kinesiophobia has been studied in musculoskeletal and neurological disorders^10,11^, its role in chronic gastrointestinal diseases such as UC has received little attention. To date, no studies have specifically analyzed kinesiophobia in patients with UC compared to healthy individuals. This is relevant because fear of movement can influence the level of physical activity and functional capacity of these patients. Furthermore, avoidance of movement can contribute to reduced mobility, gait abnormalities, and the development of secondary musculoskeletal complications.

Assessing kinesiophobia using validated instruments such as the Tampa Scale for Kinesiophobia (TSK-11) allows for the identification of movement-related fear and a better understanding of its impact on physical function. Therefore, the objective of this study was to evaluate the level of kinesiophobia in patients with UC and compare the results with those of a healthy control group using the TSK-11.

## METHODS

### Design and sample

A total of 106 participants completed this case-control study and were divided into two groups: a group with UC (n=53) and a control group of healthy subjects without UC (n=53). Subjects were recruited by consecutive sampling. The subjects with UC were recruited from the Gastroenterology Department of the Virgen Macarena University Hospital in Seville and from the Association of Patients with Crohn's Disease, Ulcerative Colitis, and Ostomies of Seville (ACCU-Seville). The healthy control subjects were recruited from a private podiatry clinic in Isla Cristina (Huelva, Spain) through consecutive sampling among individuals attending routine podiatric evaluations or preventive consultations. These participants were selected to represent community-dwelling adults without inflammatory bowel disease (IBD). The case and control groups were matched for age, sex, and body mass index (BMI).

Inclusion criteria were age between 18 and 88 years, a diagnosis of UC (case group), and no diagnosis of IBD (control group). Additionally, participants were required to be able to walk independently and to provide written informed consent in order to participate in the study. Exclusion criteria included being outside the established age range; having a diagnosis of digestive disease other than UC (case group); history of trauma and foot surgery; inability to independently perform activities of daily living; inability to understand the study instructions; and refusal to sign the informed consent, as these factors may affect functional capacity and interfere with the accurate assessment of kinesiophobia, consistent with previous case-control studies using the TSK-11^10,11^.

### Procedure

A single researcher performed all measurements. First, clinical data and sample characteristics were collected via interview, including age, sex, time since UC diagnosis, treatment, weight, height, occupation, physical activity, and comorbidities. Subsequently, participants completed the Spanish version of the TSK-11, which consists of 11 items scored from 1 to 4, where 1 (strongly disagree) and 4 (strongly agree). The total scale score ranges from 11 to 44, with higher scores corresponding to greater levels of kinesiophobia^12^. The Spanish version of the TSK-11 has strong psychometric properties (Cronbach's α =0.78; intraclass correlation coefficient=0.82)^13^.

### Ethical considerations

To conduct this study, approval was obtained from the Research Ethics Committee of the Virgen Macarena/Virgen del Rocío University Hospital in Seville (SICEIA-2025-000733). Before the research began, all participants were informed about the nature of the study and provided written informed consent. Data confidentiality and anonymity were guaranteed at all times, in compliance with Organic Law 3/2018, of December 5, on the Protection of Personal Data and the guarantee of digital rights. The principles established in the Declaration of Helsinki^14^ were also respected.

### Sample size calculation

To calculate the sample size, the G*Power software, version 3.1.9.3 (Heinrich Heine University of Düsseldorf, Germany), was used, with an effect size of 0.5, an α error of 0.05, and a statistical power (1- β) of 0.80. As a result, a total of 106 individuals were obtained for the study, which is equivalent to 53 people per group.

### Statistical analysis

Statistical analysis was performed using IBM Statistical Package for the Social Sciences version 29.0.01.0 (Armonk, NY, USA). Initially, the Kolmogorov-Smirnov test was used to determine whether the variables followed a normal distribution. For subsequent comparisons between groups, the Mann-Whitney U test was used for continuous variables and the chi-square test for categorical variables. For continuous variables, data are presented as mean ± standard deviation (SD); categorical variables are presented as number of cases and percentage. A 95% confidence level was used, and a p<0.05 was considered statistically significant in all analyses.

## RESULTS

### Descriptive data

A total of 106 subjects participated in the study. One group consisted of subjects diagnosed with UC (n=53), and the other group consisted of healthy subjects for the control (n=53). The mean age was 46.13 years (SD: 17.34), with ages ranging from 18 to 80 years. Of the participants, 50 (47.2%) were men and 56 (52.8%) were women. Table 1 presents the sociodemographic and descriptive characteristics of the sample, where it can be seen that the subjects did not show significant differences (p>0.05) between groups.

**Table 1:** Sociodemographic and descriptive data of the sample.

Descriptive data	Total sample (n=106)	UC (n=53)	Control (n=53)	p-value*
	Mean (SD)	Mean (SD)	Mean (SD)	
Age (years)	46.13 ± 17.34 (18–80)	48.70 ± 13.60 (20–79)	43.57 ± 20.22 (18–80)	0.255†
Weight (kg)	72.99 ± 14.62 (45–125)	75.16 ± 16.09 (45–125)	70.81 ± 12.76 (52–115)	0.154†
Height (m)	1.70 ± 0.09 (1.54–1.93)	1.71 ± 0.06 (1.54–1.93)	1.69 ± 0.08 (1.55–1.93)	0.427†
BMI (kg/m^2^)	25.21 ± 4.46 (17.78–42.25)	25.79 ± 5.01 (17.78–42.25)	24.65 ± 3.80 (18.34–37.55)	0.291†
Sex Male (%)	50 (47.2%)	27 (50.9%)	23 (43.4%)	0.436‡
Female (%)	56 (52.8%)	26 (49.1%)	30 (56.6%)	
Diagnosis (years)	N/A	16.08 ± 13.17 (2–50)	N/A	N/A

UC: ulcerative colitis; BMI: body mass index; M: male; F: female. In all analyses p<0.05 (with a 95%CI) was considered statistically significant.

† Mann–Whitney U test was applied.

‡ Frequencies (percentages) and chi-square (χ ^2^) test were utilized. Statistically significant differences between groups (p<0.05).

### Outcome measurements

Table 2 shows the results related to the TSK-11 kinesiophobia scores. The results present a statistically significant difference between the groups (p<0.05) in the TSK-11 total scores. Specifically, the proportion of participants with moderate to maximum kinesiophobia was substantially elevated in the UC group (49.1%) compared to the control group (30.2%). In contrast, the sum of no fear and slight fear reached only 27 cases (50.94%) for the UC group and 37 (69.81%) in the control group.

**Table 2:** Comparisons of Tampa Scale for Kinesiophobia scores and categories between ulcerative colitis and without ulcerative colitis.

Outcome measurements		Total group (n=106)	UC Mean ± SD (n=53)	Healthy Mean ± SD (n=53)	p-value (cases vs. controls)
TSK-11 category*	No	23 (21.7%)	9 (17%)	14 (26.4%)	0.153*
	Slight	41 (38.7%)	18 (34%)	23 (43.4%)	
	Moderate	28 (26.4%)	17 (32.1%)	11 (20.8%)	
	Severe	7 (6.6%)	3 (5.7%)	4 (7.5 %)	
	Maximum	7 (6.6%)	6 (11.3%)	1 (1.9%)	
TSK scores		N/A	24.98 ± 8.01 (11–44)	21.94 ± 6.18 (11–39)	**0.016** †

UC: ulcerative colitis; TSK-11: Tampa Scale Kinesiophobia; SD: standard deviation; N/A: not applicable. Frequency, percentage (%), and chi-squared test (χ ^2^) were utilized.

† Mann-Whitney U test was applied. In all the analyses, p<0.05 (with a 95%CI) was considered statistically significant (bold). Statistically significant differences between groups (p<0.05).

## DISCUSSION

The purpose of this research was to analyze and compare kinesiophobia levels between patients with UC and healthy subjects using the TSK-11 scale in order to explore whether this chronic IBD is associated with an increased fear of movement. This phenomenon has been extensively studied in musculoskeletal and systemic pathologies such as lupus erythematosus, rheumatoid arthritis^15^, fibromyalgia^16^, and multiple sclerosis^11^, but to our knowledge, no research has addressed it in chronic gastrointestinal diseases. Therefore, this research represents a novel approach to studying the psychological and functional impact of UC.

One of the strengths of this study is that the groups were matched for sex, age, and BMI, allowing for a more accurate comparison and relating the differences found to the presence of UC. Our results show that patients with UC have higher scores on the TSK-11 scale than healthy subjects, indicating a greater level of fear of movement in these patients. These results are similar to those found in other chronic diseases. Ruiz-Sánchez et al.^11^ observed that 93.1% of patients with multiple sclerosis presented with kinesiophobia and that 65.9% had moderate or severe levels. In our study, 83% of patients with UC presented with kinesiophobia, and 49.1% were at moderate or severe levels. In both cases, patients with chronic diseases showed more kinesiophobia than healthy individuals. Although multiple sclerosis and ulcerative colitis are different diseases, both present symptoms such as fatigue, pain, and functional limitations, which can cause patients to avoid physical activity. Similarly, Jiménez-Cebrián et al.^10^ found that all patients with Parkinson's disease evaluated presented with kinesiophobia and that 77.3% reached moderate or severe levels. Taken together, these results suggest that kinesiophobia is a frequent phenomenon in various chronic diseases, both of neurological and inflammatory origin.

The levels of kinesiophobia observed in patients with UC are similar to those described in other chronic diseases, suggesting that common factors may exist. Monteiro et al.^17^, in a study with lymphedema patients, found that fatigue and pain were related to the onset of fear of movement. In the case of UC, symptoms such as abdominal pain, fatigue, fecal urgency, and fear of flare-ups can cause patients to avoid movement, similar to what occurs in other chronic diseases^18-20^. However, unlike other pathologies, such as onychocryptosis, where kinesiophobia is mainly related to pain^21^, in UC, this phenomenon can be influenced by both physical and psychological factors.

Furthermore, foot problems have been associated with poorer function and a lower quality of life, especially in older adults. López López et al.^22^ observed that people with foot problems had poorer foot health and a lower quality of life than healthy individuals. This highlights the importance of foot health for physical function. In patients with ulcerative colitis, fear of movement can contribute to reduced mobility and decreased participation in physical activity. For this reason, identifying kinesiophobia can help healthcare professionals better understand these patients’ limitations and plan more appropriate treatment from both a clinical and podiatric perspective.

According to the results of this study, in clinical practice, it is important to assess kinesiophobia in patients with ulcerative colitis. Although initially, this fear of movement may appear as a response to pain or discomfort, if it persists over time, it can hinder recovery and affect quality of life^23^. Furthermore, physical activity has been shown to have positive effects in these patients, as it can improve fatigue, emotional state, and inflammation levels^24,25^. Therefore, as in other chronic diseases, early identification of fear of movement would allow for the implementation of multidisciplinary strategies aimed at promoting safety and personalized exercise routines that improve adherence to physical activity and overall disease management. From a podiatric perspective, prolonged inactivity can lead to biomechanical alterations, muscle weakness, impaired balance, and an increased risk of falls or gait disturbances^15,26^. Therefore, identifying kinesiophobia would allow podiatrists and other health professionals to design progressive exercise plans and educational interventions to improve patients’ confidence in movement, reduce sedentary behavior, and promote physical and psychological well-being.

This study has some limitations that are important to consider. As it is a cross-sectional study, we cannot definitively say that ulcerative colitis is the direct cause of kinesiophobia. Furthermore, the number of participants is small, and they all come from the same geographic area, so the results should be interpreted with caution. Future studies should include more participants from different regions and follow them for longer periods to observe how kinesiophobia evolves. It would also be useful to compare these results with those of other digestive diseases, such as Crohn's disease or irritable bowel syndrome, to determine whether the fear of movement appears in a similar way or varies depending on the disease.

## CONCLUSION

This study concludes that patients with ulcerative colitis exhibit significantly higher levels of kinesiophobia than healthy subjects, with these levels ranging from moderate to severe. Therefore, kinesiophobia should be assessed in UC patients for early detection, enabling the development of interventions to foster confidence in safe physical activity and protect and improve the patient's overall quality of life.

## Data Availability

The datasets generated and/or analyzed during the current study are available from the corresponding author upon reasonable request.
